# Blood Pressure Changes and Chemical Constituents of Particulate Air Pollution: Results from the Healthy Volunteer Natural Relocation (HVNR) Study

**DOI:** 10.1289/ehp.1104812

**Published:** 2012-10-19

**Authors:** Shaowei Wu, Furong Deng, Jing Huang, Hongyi Wang, Masayuki Shima, Xin Wang, Yu Qin, Chanjuan Zheng, Hongying Wei, Yu Hao, Haibo Lv, Xiuling Lu, Xinbiao Guo

**Affiliations:** 1 Department of Occupational and Environmental Health Sciences, Peking University School of Public Health, Beijing, China; 2 Department of Cardiology, Peking University People’s Hospital, Beijing, China; 3 Department of Public Health, Hyogo College of Medicine, Hyogo, Japan

**Keywords:** air pollution, blood pressure, hypertension, panel study, particulate matter

## Abstract

Background: Elevated blood pressure (BP) has been associated with particulate matter (PM) air pollution, but associations with PM chemical constituents are still uncertain.

Objectives: We investigated associations of BP with various chemical constituents of fine PM (PM_2.5_) during 460 repeated visits among a panel of 39 university students.

Methods: Resting BP was measured using standardized methods before and after the university students relocated from a suburban campus to an urban campus with different air pollution contents in Beijing, China. Air pollution data were obtained from central monitors close to student residences. We used mixed-effects models to estimate associations of various PM_2.5_ constituents with systolic BP (SBP), diastolic BP (DBP), and pulse pressure.

Results: An interquartile range increase of 51.2 μg/m^3^ in PM_2.5_ was associated with a 1.08-mmHg (95% CI: 0.17, 1.99) increase in SBP and a 0.96-mmHg (95% CI: 0.31, 1.61) increase in DBP on the following day. A subset of PM_2.5_ constituents, including carbonaceous fractions (organic carbon and elemental carbon), ions (chloride and fluoride), and metals/metalloid elements (nickel, zinc, magnesium, lead, and arsenic), were found to have robust positive associations with different BP variables, though robust negative associations of manganese, chromium, and molybdenum with SBP or DBP also were observed.

Conclusions: Our results support relationships between specific PM_2.5_ constituents and BP. These findings have potential implications for the development of pollution abatement strategies that maximize public health benefits.

Both elevated blood pressure (BP) and ambient particulate air pollution have been associated with increased cardiovascular morbidity and mortality (Blacher et al. 2000; Brook et al. 2010; Guo et al. 2010; Kan et al. 2007). An elevation in BP may be an important physiological mechanism linking particulate air pollution and adverse cardiovascular outcomes (Brook and Rajagopalan 2009). Among the size fractions of ambient particulate matter (PM), PM with an aerodynamic diameter ≤ 2.5 µm (PM_2.5_) has been associated with adverse cardiovascular effects (Brook et al. 2010; Kan et al. 2007). Controlled human and animal experiments have verified that exposure to PM_2.5_ is capable of inducing elevated BP (Brook et al. 2009; Chang et al. 2004; Urch et al. 2005; Zanobetti et al. 2004).

Ambient PM is a mixture of various chemical constituents, including carbonaceous fractions [organic carbon/elemental carbon (OC/EC)], ions, and transition metals. These constituents may have different effects on the cardiovascular system (Brook et al. 2010). Although several PM chemical constituents (e.g., carbonaceous fractions) have been associated with prohypertensive effects in different populations (Mordukhovich et al. 2009; Urch et al. 2005; Zanobetti et al. 2004), evidence for effects of specific PM chemical constituents on BP is still lacking. Specifically, traffic-related PM may play a distinctive role in cardiovascular responses (Auchincloss et al. 2008; Brook et al. 2009; Delfino et al. 2010; Jia et al. 2012; Wu et al. 2010, 2011a, 2011b). Thus, a hypothesis that a group of PM chemical constituents and related sources may confer greater PM cardiovascular toxicity is reasonable based on the existing literature. We conducted the Healthy Volunteer Natural Relocation (HVNR) study to examine the relationship between various PM_2.5_ chemical constituents and BP changes in a panel of healthy male university students in Beijing, China, before and after their relocation from a suburban campus to an urban campus with different PM air pollution constituents. We hypothesized that this relocation would substantially change the participants’ exposures to ambient PM_2.5_ and chemical constituents associated with local pollution sources, and thus facilitate an analysis of relationships between PM_2.5_ chemical constituents and BP in the study population.

## Material and Methods

**Figure 1:**
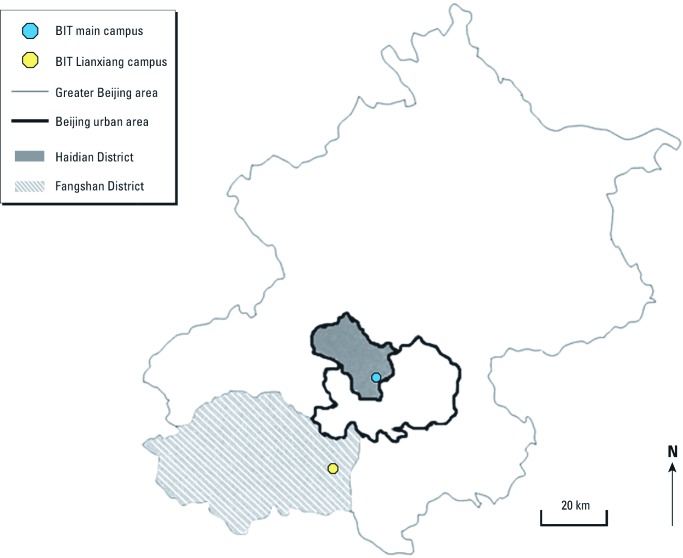
Map showing locations of the two BIT campuses involved in the study.

*Study design.* Beijing City covers an area of 16,410 km^2^, has nearly 20,000,000 inhabitants, and is about 160 km from the nearest coastline. More than 5,000,000 vehicles are its main source of urban air pollution. Our study population consisted of a panel of 41 male undergraduate college students from a university in Beijing [Beijing Institute of Technology (BIT)]. The BIT has two campuses located in different areas of Beijing (Figure 1). Study participants completed their first 2 years of undergraduate study (from autumn 2008 to summer 2010) at the BIT Liangxiang campus, which is located in a suburban area (Fangshan District), then moved to the BIT main campus, which is located in an urban area (Haidian District) for their next 2 years of study (from autumn 2010 to summer 2012). The BIT Liangxiang campus is about 2 km from the nearest freeway. There were several active construction sites within 2 km of the campus during the study, in addition to some industrial facilities located within several kilometers of the campus. In contrast, the BIT main campus is located in the Beijing downtown area, along the northwest inner side of the third ring road that circles the city. There were no substantive construction activities or industrial facilities near the main campus during the study. We used the following inclusion criteria to select participants before the study began: male with a geographical origin other than Beijing, non-obese, no history of smoking, and without pulmonary, cardiovascular, and other chronic diseases. We used a self-administered questionnaire to collect personal information, including name, age, and medical history/health status. We scheduled 12 biweekly study visits for each participant over the entire study, including 4 visits during each of the following three time periods: suburban period (22 April to 20 June 2010) at the BIT Liangxiang campus and urban period 1 (3 September to 8 November 2010) and urban period 2 (10 April to 12 June 2011) at the BIT main campus. The study was approved by the Institutional Review Board of Peking University Health Science Center, and informed consent was provided by each participant before the study began.

*BP measurement.* Study visits were scheduled between 1300 and 1500 hours on weekdays to minimize diurnal variation in BP. A trained technician in the hospital affiliated with the BIT Liangxiang campus or BIT main campus performed standardized resting BP measurements during each study visit. Participants rested in a sitting position in a quiet room for at least 10 min before upper arm BP was measured using an Omron 705IT automated oscillometric monitor (HEM-759-E; Omron Healthcare Co. Ltd., Kyoto, Japan) at least three times with a 1-min minimum interval between measurements. In most cases the second and third sets of readings were averaged to calculate systolic BP (SBP) and diastolic BP (DBP) (Rioux et al. 2010). However, if the difference between SBP or DBP values of the second and third measurements was > 5 mmHg, the BP was considered unstable, and another 1 to 3 measurements would be taken until the difference between the last two measurements was ≤ 5 mmHg. Under this condition, all readings (from the second to the last measurement) within a 5-mmHg range of difference were averaged to calculate the final BP values. Pulse pressure (PP) was calculated as the difference between the average SBP and DBP values. Weight and height were measured during the first study visit in each time period, and body mass index (BMI) was calculated as weight in kilograms divided by height in meters squared. Two volunteers who did not complete study visits after relocation to the urban campus were excluded from data analysis, leaving a total of 39 participants.

*Environmental data.* Air pollution and weather data were measured using standard methods and quality controls at a central monitoring site on the BIT Liangxiang campus (suburban period) or the BIT main campus (urban periods 1 and 2). The BIT Liangxiang monitoring site was on the roof of a three-story building (about 10 m high) without any nearby structures that would obstruct air flow, and the BIT main campus monitoring site was on the roof of a five-story building (about 15 m high) located within 200 m of the third ring road. The instruments and materials used for air monitoring included SKC sampling systems for PM_2.5_ mass collection on Teflon filters and quartz-fiber filters (SKC Inc., Eighty Four, PA, USA) and a digital dust monitor for real-time PM_2.5_ concentration measurement (LD-3K; Sibata Scientific Technology Inc., Tokyo, Japan); a model T15n enhanced carbon monoxide (CO) measurer for real-time CO concentration measurement (Langan Products Inc., San Francisco, CA, USA); Ogawa passive samplers for nitrogen oxides and nitrogen dioxide (NO_x_ and NO_2_, respectively) collection on cellulose fiber filters (Ogawa Air Inc., Osaka, Japan); and a HOBO Pro V2 logger for temperature and relative humidity measurements (Onset Corp., Pocasset, MA, USA). Data on PM with an aerodynamic diameter of ≤ 10 µm (PM_10_) were obtained from the nearest city air monitoring stations (within 5 km of each campus) under the supervision of the Beijing Municipal Environmental Protection Bureau. Concentrations of coarse PM with an aerodynamic diameter of between 2.5 and 10 μm (PM_2.5–10_) were calculated as the difference between the measured PM_10_ and PM_2.5_ concentrations.

Daily PM_2.5_ mass concentrations were determined by standard weighing procedures before and after the sample collection (Wu et al. 2010). The PM_2.5_ filters were analyzed in the laboratory for the following chemical constituents: OC and EC in quartz-fiber filters by thermo/optical transmission method (Lab OC/EC analyzer; Sunset Laboratory Inc., Tigard, OR, USA); sulfate, nitrate, chloride, and fluoride in Teflon filters by ion chromatography (model ICS-2000; Dionex Corp., Sunnyvale, CA, USA); aluminum, calcium, sodium, potassium, magnesium, iron, and zinc in Teflon filters by inductively coupled plasma atomic emission spectrometry (model SPS8000; KCHG Co. Ltd., Beijing, China); and strontium, barium, lead, copper, titanium, nickel, molybdenum, cadmium, vanadium, chromium, manganese, arsenic, selenium, stannum, and antimony in Teflon filters by inductively coupled plasma mass spectrometry (model ELAN DRC II; PerkinElmer Inc., Shelton, CT, USA). We also estimated the concentrations of three additional carbonaceous fractions [primary OC (POC), secondary OC (SOC), and particulate organic matter (POM)], as described in detail in Supplemental Material, p. 2 (http://dx.doi.org/10.1289/ehp1104812). PM_2.5_ constituents were classified according to their chemical nature as carbonaceous fractions, negative ions, transition metals, crustal metals, or other metals/metalloid elements. NO_x_ and NO_2_ were collected on cellulose fiber filters and concentrations were determined using a spectrophotometer following the manufacturer’s specifications (Ogawa & Company USA, Inc., Pompano Beach, FL, USA). Nitric oxide (NO) concentrations were calculated as the difference between the NO_x_ and NO_2_ concentrations.

*Statistical analysis.* We first used paired *t*-tests to compare the mean BP changes between periods by subject, and then we used mixed-effects regression models in SAS version 9.2 (SAS Institute Inc., Cary, NC, USA) to estimate associations between exposure variables and BP. Environmental data were matched with BP data for each subject before analysis. The mixed-effects models included a random intercept for each subject to account for within-subject correlations due to repeated measurements. Base models included individual air pollutants or PM_2.5_ constituents, and were adjusted for age, BMI, temperature, and relative humidity as continuous variables, with linear and quadratic terms for temperature and relative humidity (Mordukhovich et al. 2009), and also adjusted for season, month, day-of-week, hour-of-day, and study site as binary or categorical variables. In addition, we included a day-of-study variable and a squared day-of-study variable in the models to account for secular trends in associations between air pollution and BP (Penttinen et al. 2001).

We used three kinds of models to investigate the associations between exposure variables and BP after combining the data over the three time periods. First, we modeled individual air pollutants or PM_2.5_ constituents to estimate associations with BP, with adjustment for the potential confounders listed above. Second, we estimated associations of individual PM_2.5_ constituents and BP with adjustment for total PM_2.5_. Third, we used a constituent residual model analysis to address collinearity between total PM_2.5_ and PM_2.5_ constituents (Wu et al. 2011a). Specifically, we regressed daily concentrations of each PM_2.5_ constituent on total PM_2.5_ concentrations using a separate linear regression model for each time period to generate a constituent residual for each daily concentration value of the constituent. The constituent residual represented the proportion of the constituent that is uncorrelated with total PM_2.5_ and therefore can be interpreted as a measure of the independent contribution of each constituent to associations with BP.

To estimate the cumulative effects of exposure, we modeled the mean concentrations of exposure variables during the preceding 1–5 days before BP measurement (Hoffmann et al. 2012). We reported results using mean concentrations during the preceding 1–3 days because most associations with BP were observed with exposures during this time period. Results are expressed as absolute changes [in millimeters mercury (mmHg)] with 95% CIs for the BP variables associated with interquartile range (IQR) increases in air pollutants and PM_2.5_ constituents. The statistical significant level was defined as *p* < 0.05 (two sided).

## Results

The mean (range) age of the eligible study subjects (*n* = 39) was 20.1 years (19–22 years), and their mean (range) BMI was 21.2 kg/m^2^ (17.2–24.9 kg/m^2^). Overall, 34 subjects completed all 12 biweekly visits, 4 completed 11 visits, and 1 completed 8 visits, resulting in a total of 460 visits. SBP and PP increased over the three time periods, whereas DBP levels remained relatively stable (Table 1).

**Table 1:** Descriptive statistics on BP by time period.

Variable/period	*n*	Mean ± SD (mmHg)	Range (mmHg)	p-Value^a^
SBP	
Suburban period	153	117.0 ± 10.1	91–146
Urban period 1	156	119.1 ± 11.4	90–154
Urban period 2	151	120.9 ± 12.1	89–156
Total	460	119.0 ± 11.3	89–156	0.010
DBP	
Suburban period	153	65.8 ± 7.3	41–91
Urban period 1	156	66.2 ± 6.9	54–94
Urban period 2	151	65.9 ± 6.7	54–89
Total	460	66.0 ± 7.0	41–94	0.847
PP	
Suburban period	153	51.2 ± 8.3	29–75
Urban period 1	156	52.9 ± 7.9	32–71
Urban period 2	151	55.0 ± 9.3	32–73
Total	460	53.0 ± 8.7	29–75	< 0.001
ap-Value for analysis variance for repeated measurements.					

Most air pollutants and PM_2.5_ constituents showed substantial variation over the three time periods (Table 2). In particular, concentrations of gaseous air pollutants (e.g., CO, NO_x_, and NO_2_) and levels of several PM_2.5_ carbonaceous fractions related to traffic (OC, EC, POC, and POM based on concentrations or proportions of PM_2.5_ mass) were higher during the urban periods than in the suburban period.

**Table 2:** Descriptive statistics on daily environmental variables over the study.

Variable	Suburban period		Urban period 1		Urban period 2		IQR^a^
	Mean ± SD	Percent of PM_2.5_	Mean ± SD	Percent of PM_2.5_	Mean ± SD	Percent of PM_2.5_	
PM10 (μg/m3)	135.4 ± 64.1		111.3 ± 75.0		129.4 ± 87.2		66.0
PM2.5–10 (μg/m3)	56.1 ± 47.5		36.3 ± 29.3		69.5 ± 62.8		42.3
PM2.5 (μg/m3)	82.0 ± 46.6		78.1 ± 72.5		59.9 ± 40.3		51.2
Carbonaceous fractions														
OC (μg/m3)	10.2 ± 5.5	15.5	12.6 ± 9.0	20.6	10.6 ± 3.9	24.0	3.7
EC (μg/m3)	2.0 ± 1.2	2.88	3.3 ± 2.6	4.65	1.7 ± 0.9	3.64	1.5
POC (μg/m3)	4.4 ± 2.3	6.26	8.2 ± 6.3	11.4	6.0 ± 3.1	13.2	4.1
SOC (μg/m3)	5.9 ± 4.9	9.30	4.4 ± 3.3	9.20	4.6 ± 2.4	10.8	1.9
POM (μg/m3)	16.4 ± 8.8	24.9	20.1 ± 14.4	32.9	16.9 ± 6.2	38.4	5.9
Ions
SO42– (μg/m3)	16.8 ± 15.0	18.4	12.0 ± 14.2	13.0	8.9 ± 9.0	13.1	10.0
NO3– (μg/m3)	2.9 ± 2.8	2.98	3.5 ± 4.7	3.21	2.1 ± 2.8	2.57	2.6
Cl– (μg/m3)	1.0 ± 1.1	1.18	1.5 ± 1.8	1.73	0.9 ± 1.2	1.40	1.2
F– (ng/m3)	31.9 ± 32.3	0.052	57.9 ± 48.2	0.107	51.7 ± 47.1	0.099	57.0
Transition metals														
Fe (μg/m3)	0.8 ± 0.4	1.21	0.7 ± 0.4	1.16	0.8 ± 0.9	1.30	0.5
Zn (μg/m3)	0.4 ± 0.3	0.52	0.4 ± 0.4	0.50	0.4 ± 0.3	0.62	0.3
Mn (ng/m3)	65.9 ± 45.0	0.116	60.4 ± 30.6	0.110	48.3 ± 26.9	0.096	35.5
Ti (ng/m3)	48.5 ± 33.3	0.071	38.0 ± 24.2	0.068	52.6 ± 79.8	0.093	29.5
Cu (ng/m3)	30.1 ± 24.3	0.038	37.5 ± 35.4	0.049	26.3 ± 22.9	0.042	26.9
Cr (ng/m3)	18.5 ± 22.5	0.036	8.9 ± 5.6	0.019	8.5 ± 6.7	0.021	5.1
Ni (ng/m3)	3.7 ± 2.2	0.006	3.4 ± 3.5	0.006	3.3 ± 2.5	0.007	2.1
Cd (ng/m3)	2.6 ± 2.8	0.003	3.2 ± 3.4	0.004	2.4 ± 2.7	0.004	2.5
V (ng/m3)	3.1 ± 1.8	0.004	1.6 ± 1.1	0.003	1.7 ± 1.9	0.003	1.3
Mo (ng/m3)	1.9 ± 2.1	0.004	1.3 ± 0.8	0.002	0.8 ± 0.7	0.002	0.9
Crustal metals
K (μg/m3)	1.1 ± 0.9	1.32	1.4 ± 0.9	2.59	1.0 ± 0.9	1.71	1.0
Ca (μg/m3)	1.1 ± 0.8	1.64	0.8 ± 0.4	1.61	1.0 ± 0.7	1.79	0.7
Al (μg/m3)	1.0 ± 0.8	1.46	0.5 ± 0.2	0.97	0.8 ± 1.6	1.23	0.5
Na (μg/m3)	0.6 ± 0.6	0.82	0.8 ± 0.5	1.60	0.6 ± 0.4	1.46	0.4
Mg (ng/m3)	292.4 ± 249.1	0.43	194.2 ± 98.9	0.37	318.4 ± 341.6	0.57	148.3
Ba (ng/m3)	14.0 ± 8.2	0.021	17.2 ± 10.0	0.033	14.6 ± 12.4	0.027	11.8
Sr (ng/m3)	9.5 ± 6.4	0.015	6.0 ± 3.1	0.011	6.6 ± 5.0	0.012	4.8
Other metals/metalloid elements														
Pb (ng/m3)	127.7 ± 89.6	0.158	125.0 ± 122.5	0.153	119.2 ± 111.7	0.181	127.7
As (ng/m3)	16.0 ± 19.9	0.018	17.1 ± 24.0	0.018	32.6 ± 35.2	0.058	19.5
Sn (ng/m3)	7.3 ± 5.7	0.009	9.2 ± 9.5	0.012	6.8 ± 5.8	0.011	6.8
Sb (ng/m3)	6.6 ± 5.6	0.008	8.6 ± 7.6	0.011	6.0 ± 4.9	0.010	6.1
Se (ng/m3)	7.3 ± 5.9	0.009	5.4 ± 4.6	0.008	3.9 ± 3.4	0.006	4.4
CO (ppm)	0.90 ± 0.33		1.67 ± 0.60		1.47 ± 0.45		0.74
NOx (ppb)	41.6 ± 18.1		70.7 ± 39.4		48.3 ± 17.7		35.1
NO2 (ppb)	24.1 ± 8.0		38.0 ± 14.8		31.7 ± 11.8		14.2
NO (ppb)	17.4 ± 12.4		32.8 ± 29.3		16.5 ± 10.9		26.8
Temperature (°C)	23.8 ± 5.3		17.3 ± 6.5		22.4 ± 4.5		—
Relative humidity (%)	47.5 ± 15.9		55.7 ± 15.7		37.9 ± 14.5		—
Abbreviations: Al, aluminum; As, arsenic; Ba, Barium; Ca, calcium; Cd, cadmium; Cl–, chloride; CO, carbon monoxide; Cr, chromium; Cu, copper; EC, elemental carbon; F–, fluoride; Fe, iron; K, potassium; Mg, magnesium; Mn, manganese; Mo, molybdenum; Na, sodium; Ni, nickel; NO, nitric oxide; NO3–, nitrate; NOx, nitrogen oxides; OC, organic carbon; Pb, lead; POC, primary organic carbon; POM, particulate organic matter; Sb, antimony; Se, selenium; Sn, stannum; SO42–, sulfate; SOC, secondary organic carbon; Sr, strontium; Ti, titanium; V, vanadium; Zn, zinc. aIQR values of air pollutant concentrations during the preceding 1 day before the BP measurement.														

SBP and PP levels during the urban periods were significantly higher than during the suburban period, and SBP and PP levels during the urban period 2 were also significantly higher than those during the urban period 1 (Table 3). There was no significant difference in DBP levels between any two periods. IQR increases in major air pollutant concentrations (PM fractions, NO_x_, and NO_2_) during the 1–3 days before study visits showed significant positive associations with SBP or DBP, with more consistent associations observed with DBP than SBP [see Supplemental Material, Table S1 (http://dx.doi.org/10.1289/ehp1104812)]. An IQR increase (51.2 μg/m^3^) in PM_2.5_ during the preceding day was associated with a 1.08-mmHg (95% CI: 0.17, 1.99) increase in SBP and a 0.96-mmHg (95% CI: 0.31, 1.61) increase in DBP. There were no significant associations between the major air pollutants and PP (data not shown).

**Table 3:** Mean BP changes (mmHg) between periods by subject.

Period pair/variable	n (subjects)^a^	Mean ± SD^b^	Range	p-Value^c^
Urban period 1–suburban period
SBP	36	2.2 ± 5.6	–11.8–12.8	0.024
DBP	36	0.4 ± 3.3	–7.3–9.9	0.472
PP	36	1.8 ± 4.7	–11.2–12.7	0.026
Urban period 2–suburban period
SBP	34	3.5 ± 5.3	–8.2–15.9	0.001
DBP	34	–0.1 ± 2.1	–3.9–4.6	0.711
PP	34	3.7 ± 5.1	–6.0–13.7	< 0.001
Urban period 2–urban period 1
SBP	37	1.7 ± 4.6	–9.8–13.2	0.035
DBP	37	–0.4 ± 3.3	–8.1–6.4	0.291
PP	37	2.1 ± 3.8	–9.4–9.1	0.001
aEach comparison is restricted to the subjects who had completed all eight study visits over the two periods being compared. bMean difference between the two periods (the latter period of the pair is used as the reference). cPaired t‑test.								

**Figure 2:**
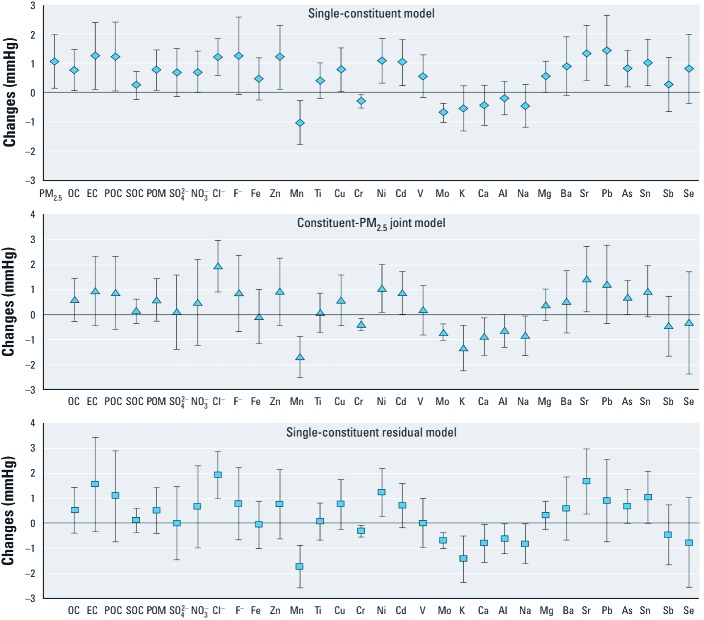
Changes in SBP associated with IQR increases in PM_2.5_ constituents at concentration during the preceding day before the BP measurement. Estimates are adjusted for age, BMI, season, month, day-of-study, squared day-of-study, day-of-week, hour-of-day, study site, temperature, and relative humidity in linear and quadratic terms. Data are presented as effect estimates ± 95% CIs. For constituent-PM_2.5_ joint models, we used the main effect estimates of PM_2.5_ constituents for result presentation.

**Figure 3:**
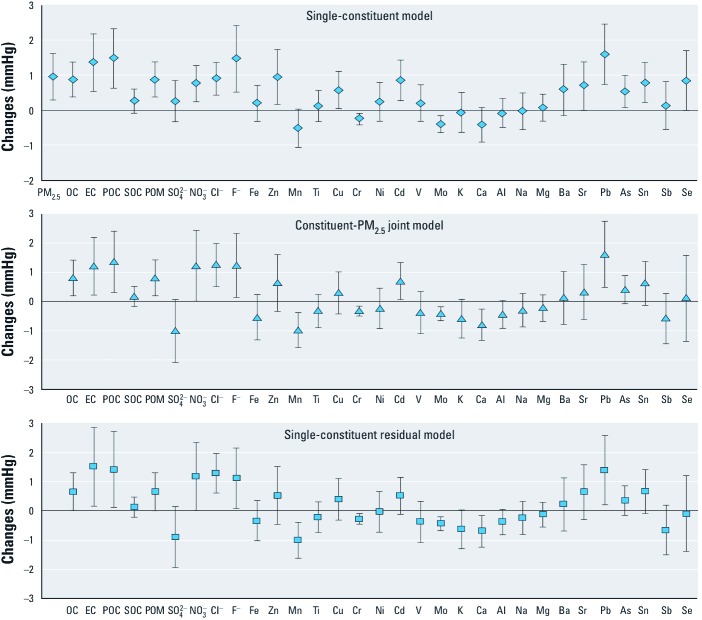
Changes in DBP associated with IQR increases in PM_2.5_ constituents at concentration during the preceding day before the BP measurement. Estimates are adjusted for age, BMI, season, month, day-of-study, squared day-of-study, day-of-week, hour-of-day, study site, temperature, and relative humidity in linear and quadratic terms. Data are presented as effect estimates ± 95% CIs. For constituent-PM_2.5_ joint models, we used the main effect estimates of PM_2.5_ constituents for result presentation.

**Figure 4:**
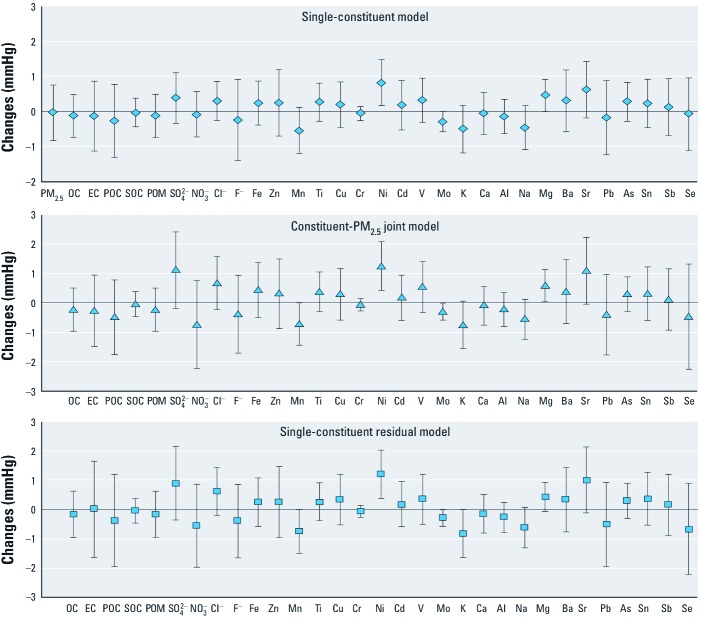
Changes in PP associated with IQR increases in PM_2.5_ constituents at concentration during the preceding day before the BP measurement. Estimates are adjusted for age, BMI, season, month, day-of-study, squared day-of-study, day-of-week, hour-of-day, study site, temperature, and relative humidity in linear and quadratic terms. Data are presented as effect estimates ± 95% CIs. For constituent-PM_2.5_ joint models, we used the main effect estimates of PM_2.5_ constituents for result presentation.

We found significant associations between SBP or DBP and concentrations of several PM_2.5_ constituents during the preceding day, though some associations that were statistically significant based on single-constituent models were not significant after adjustment for PM_2.5_ or based on constituent residual models (Figures 2–4). However, all three model estimates indicated significant positive associations between SBP and chloride, nickel, and strontium (Figure 2); between DBP and OC, EC, POC, POM, chloride, fluoride, and lead (Figure 3); and between PP and nickel and magnesium (Figure 4). In addition, all three model estimates indicated significant negative associations between SBP and manganese, chromium, and molybdenum, and between DBP and chromium and molybdenum. We also found significant associations between SBP and zinc during the preceding 3 days, and between PP and arsenic during the preceding 3 days based on three different models (data available upon request).

## Discussion

We evaluated relationships between BP and various air pollutants and PM_2.5_ chemical constituents under natural exposure settings using a panel study design that repeatedly measured resting BP during three time periods with different ambient air pollution exposures. The relocation of the study population from a suburban campus to an urban campus, which was a normal part of their university education, gave us the opportunity to study associations with specific PM_2.5_ constituents that may have distinct cardiovascular effects (Brook et al. 2009). We observed increases in SBP and PP after relocation from the suburban campus to the urban campus located in a megacity with high air pollution levels, and we estimated consistent associations between BP measures and a subset of PM_2.5_ chemical constituents.

Major sources of particulate air pollution in Beijing include road dust, motor vehicle exhaust, industry, incineration, and coal burning (Sun et al. 2004). During recent years, the number of motor vehicles in Beijing has increased rapidly and traffic emissions have become a dominant source of ambient air pollution (Stone 2008; Wang et al. 2009; Zhou et al. 2010). Evidence linking traffic-related air pollution and cardiovascular outcomes has been growing, especially in urban areas where traffic emissions are one of the major pollution sources (Brook et al. 2009; Delfino et al. 2010; Jia et al. 2012; Wu et al. 2010, 2011a, 2011b). OC and EC (or its surrogate, black carbon) are two commonly used indicators of traffic emissions (Delfino et al. 2010; Sun et al. 2004; Wang et al. 2009). Short-term exposures to these carbonaceous particles have been associated with prohypertensive effects in patients with cardiovascular conditions (Delfino et al. 2010; Zanobetti et al. 2004). In our study, we found that PM_2.5_ measured in the urban area contained larger proportions of carbonaceous fractions than PM_2.5_ measured in the suburban area (Table 2). This suggests a greater contribution of traffic emissions to the particulate air pollution in the urban area. We found robust positive associations between PM_2.5_ carbonaceous fractions and DBP in our study participants. When OC was subclassified as POC or SOC, the association appeared to be specific to POC (Figure 3). POC is a representative indicator of particulate organics from fossil-fuel combustion sources in the context of traffic-related air pollution (Delfino et al. 2010). Therefore, our findings suggest that PM carbonaceous fractions related to traffic may play an important role in the prohypertensive effects of PM air pollution.

Several PM_2.5_ metal constituents, including nickel, zinc, magnesium, strontium, lead, and arsenic, had robust positive associations with SBP, DBP, or PP, whereas manganese, chromium, and molybdenum had robust negative associations with SBP or DBP. Among these constituents, nickel, zinc, manganese, chromium, and lead typically come from industrial emissions, including metallurgical processes, but some of these metals (e.g., zinc, nickel, manganese) may also come from traffic emissions (Loranger and Zayed 1995; Sun et al. 2004). Magnesium usually originates from mineral aerosols that may result from resuspended road dust and long-range transported dust (Sun et al. 2004), and arsenic is typically generated from coal burning (Xie et al. 2006). Relationships between BP and these metals/metalloid elements, especially the transition metals (e.g., nickel, zinc, manganese, cadmium), have been well demonstrated in previous toxicological studies *in vivo* (Fiorim et al. 2011; Kasten et al. 1994; Wang et al. 2002; Yanagisawa et al. 2004; Yang et al. 2007). When delivered to the airways, the transition metals could stimulate the production of reactive oxygen species and then induce airway injury and inflammation, which are subsequently followed by a series of cardiopulmonary responses (González-Flecha 2004). Epidemiologic evidence linking metals/metalloid elements and BP has also been growing. For example, chronic exposures to arsenic and lead have been associated with increased BP or higher hypertension prevalence in populations (Mordukhovich et al. 2012; Navas-Acien et al. 2007). Specifically, we found robust negative associations between manganese, chromium, and molybdenum and SBP or DBP. Environmental manganese is generally considered to reduce hypertension risk (Houtman 1996). A previous animal experiment found that infusing manganese into conscious, restrained rats resulted in a decrease in BP (Kasten et al. 1994), and results from a recent epidemiologic study found negative associations between chronic manganese exposure and BP in elderly men (Mordukhovich et al. 2012). However, our findings on the negative associations between BP and chromium and molybdenum are not supported by previous experimental and epidemiologic reports, and a causal explanation for these findings may require further investigation.

We found small but robust positive associations between chloride and SBP and DBP, less consistent associations between fluoride and DBP, and inconsistent associations between sulfate and nitrate and BP. Sulfate and nitrate are typical secondary pollutants that constitute a significant proportion of the PM_2.5_ mass. In urban areas far from coastlines, the major source for airborne chloride may be from burning polyvinylchloride plastic in refuse dumps, and large amounts of pollutants such as fluoride may also be emitted when trash is being smashed or incinerated (Sun et al. 2004). Experimental studies have demonstrated that chloride was critical in the development of hypertension (Kurtz and Morris 1984; Kurtz et al. 1987; Ziomber et al. 2008). That is, dietary intake of several ions, including sodium, potassium, and chloride, were able to induce increased BP in rats (Ziomber et al. 2008), whereas sodium loading without chloride failed to increase BP in animals (Kurtz and Morris 1984) or men (Kurtz et al. 1987). Nevertheless, evidence for the relationship between these airborne ions and BP is still rare and requires further investigation.

Associations with the different BP variables differed over the study. SBP and PP levels tended to increase over time, whereas DBP levels did not. Among the BP variables, PP has been regarded as a stronger predictor of adverse cardiac outcomes, especially in hypertensive patients (Blacher et al. 2000). Several studies have investigated associations between air pollution and BP, but only a few of them have examined PP (Auchincloss et al. 2008; Rioux et al. 2010). Our findings also indicated wide variation among individual study participants, with SBP or PP increasing by > 10 mmHg after relocation to the urban campus in some cases. This suggests that susceptibility to the effects of air pollutants may vary substantially among individuals in the general population.

The study has several strengths in addition to the natural relocation study design. Participants were young, healthy volunteers who were nonsmokers and free of any cardiovascular compromises. Therefore, confounding by factors such as age, smoking, disease status, medication use, or obesity was unlikely. We conducted the study in the spring and autumn seasons to avoid significant climate changes that might confound associations between air pollutants and BP (Adamopoulos et al. 2010). We used constituent residual models to estimate associations with PM_2.5_ constituents, and we adjusted for many potential confounders. However, residual or unmeasured confounding cannot be excluded, and generalizability to other populations may be limited.

This study has several other limitations. First, we used ambient air pollution data from central monitoring sites rather than personal exposure measures. However, all participants lived in school dormitories within 300 m of the monitoring site for each campus, and they spent most of their time in naturally ventilated buildings near the monitoring site. Therefore, air pollution data from central monitoring sites could be used as good surrogates for their real exposures. Second, as in most epidemiologic studies, we were not able to determine whether the observed associations were due to the measured air pollution constituents or to other factors that varied along with constituent concentrations (e.g., pollution sources, seasons, locations, or even other pollutants that might be related or correlated to the measured constituents). For example, both seasonal and regional variation in estimated effects of air pollution on cardiovascular outcomes have been reported by previous studies (Brook et al. 2009; Choi et al. 2007). Changes in season or location may result in changes in air pollution sources and the constituents of air pollution, which in turn may influence the effects of air pollution on the cardiovascular system. As a result, we were not able to differentiate effects of season or location from potential effects of air pollution in the current study. Third, other gaseous air pollutants that may also contribute to the adverse cardiovascular outcomes (e.g., ozone and sulfur dioxide) were not measured in the present study. Finally, there is a potential confounding effect associated with the progression in university education on BP through changes in the participants’ stress levels across different periods that could not be excluded. However, in view that the estimated air pollution effects were quite strong after adjusting for various factors related to seasonal and regional factors, we believe this kind of confounding effect would not be able to change our findings materially.

## Conclusions

Our findings suggest that specific PM_2.5_ chemical constituents may be associated with BP in healthy adults. These findings also suggest potential linkages between pollution sources and PM-related cardiovascular effects. A better understanding of the responsible PM constituents and their sources could lead to more targeted and effective regulations (Brook et al. 2010). This is especially important for the most polluted regions and countries around the world. As the largest developing country, China is now facing the worst air pollution problem in the world along with its rapid economic expansion over the past decades (Kan et al. 2012). Our findings thus may have implications for the development of relevant pollution abatement strategies that maximize benefits to public health.

## Supplemental Material

(53 KB) PDF
