# Supplementary material for: Blood Pressure Changes and Chemical Constituents of Particulate Air Pollution: Results from the Healthy Volunteer Natural Relocation (HVNR) Study
Source: Environ Health Perspect. 2012 Oct 19;121(1):66–72. doi: 10.1289/ehp.1104812 (PMC3546346; doi:10.1289/ehp.1104812)
Supplement: (53 KB) PDF [file ehp.1104812.s001.pdf]

Supplemental Material

Blood Pressure Changes and Chemical Constituents of Particulate Air Pollution: Results From The Healthy Volunteer Natural Relocation (HVNR) Study

Shaowei Wu, Furong Deng, Jing Huang, Hongyi Wang, Masayuki Shima, Xin Wang, Yu Qin, Chanjuan Zheng, Hongying Wei, Yu Hao, Haibo Lv, Xiuling Lu, Xinbiao Guo

Table of Contents

Supplemental Methods: p.2
References: p.3

Supplemental Material, Table S1. BP changes (mmHg) and 95% CIs associated with IQR increases in major air pollutants at concentrations during the preceding 1 to 3 days before the BP measurement: p.4

Supplemental Methods

Several additional carbonaceous fractions, including secondary OC (SOC), primary OC (POC), and particulate organic matter (POM), denote different components of organic constituents within particles. The OC can be either released directly into the atmosphere (POC) or produced from gas-to-particle reactions (SOC) (Feng et al. 2009). These constituents may have different potentials to affect the cardiovascular health as partly demonstrated in a few recent studies (Delfino et al. 2009, 2010).

The contributions of SOC and POC to measured ambient OC were estimated from OC and EC concentrations using EC as a tracer of primary combustion generated OC (i.e., “EC tracer method”) (Castro et al. 1999; Duan F et al. 2005; Turpin and Huntzicker 1995). A previous study suggested using the minimum OC/EC ratios of the ambient aerosols as primary OC/EC ratios and calculating SOC assuming that the meteorological conditions are not favorable for the SOC formation in some cases (Castro et al. 1999). These cases include the lack of direct solar radiation, low ozone concentration and unstable air mass (Dusek 2000). This method has been successfully applied in several previous studies estimating the SOC concentrations in Chinese urban areas (Cao et al. 2004; Duan F et al. 2005; Duan J et al. 2007; Feng et al. 2009). In our study, we thus estimated the SOC concentrations by the following expression:

$$SOC = OC_{tot} - EC \times (OC/EC)_{min} \quad (1)$$

where SOC is the secondary organic carbon, OC_{tot} the total measured ambient organic carbon, and $(OC/EC)_{min}$ the minimum OC/EC ratio of the ambient aerosols. To account for heterogeneous relationships between OC and EC over different periods, we used the minimum OC/EC ratio of each time period to estimate the SOC concentrations for that time period. The POC concentrations were calculated as the differences between OC_{tot} and SOC concentrations.

In addition, we also estimated the concentrations of particulate organic matter (POM). POM can be estimated by multiplying the measured OC by a factor to compensate for other atoms such as H, O and N in the organic molecule (Duan F et al. 2005). A previous study suggested a factor of 1.6 for the estimation of POM in urban aerosols (Turpin and Lim 2001), and this factor has also been successfully applied in several studies in Chinese urban areas (Duan F et al. 2005, Yang et al. 2005). Therefore the factor of 1.6 was adopted in the current study.

References

Cao JJ, Lee SC, Ho KF, Zou SC, Fung K, Li Y, et al. 2004. Spatial and seasonal variations of atmospheric organic carbon and elemental carbon in Pearl River Delta Region, China. Atmos Environ 38:4447-4456.

Castro LM, Pio CA, Harrison RM, Smith DJT. 1999. Carbonaceous aerosol in urban and rural European atmospheres: estimation of secondary organic carbon concentrations. Atmos Environ 33;2771-2781.

Delfino RJ, Staimer N, Tjoa T, Gillen DL, Polidori A, Arhami M, et al. 2009. Air pollution exposures and circulating biomarkers of effect in a susceptible population: clues to potential causal component mixtures and mechanisms. Environ Health Perspect 117:1232-1238.

Delfino RJ, Tjoa T, Gillen DL, Staimer N, Polidori A, Arhami M, et al. 2010. Traffic-related air pollution and blood pressure in elderly subjects with coronary artery disease. Epidemiology 21:396-404.

Duan F, He K, Ma Y, Jia Y, Yang F, Lei Y, et al. 2005. Characteristics of carbonaceous aerosols in Beijing, China. Chemosphere 60:355-364.

Duan J, Tan J, Cheng D, Bi X, Deng W, Sheng G, et al. 2007. Sources and characteristics of carbonaceous aerosol in two largest cities in Pearl River Delta Region, China. Atmos Environ 41:2895-2903.

Dusek U. Secondary Organic Aerosol—Formation Mechanisms and Source Contributions in Europe. (www.iiasa.ac.at) 2000. Interim Report IR-00-066.

Feng Y, Chen Y, Guo H, Zhi G, Xiong S, Li J, et al. 2009. Characteristics of organic and elemental carbon in $PM_{2.5}$ samples in Shanghai, China. Atmos Res 92:434-442.

Turpin BJ, Huntzicker JJ. 1995. Identification of secondary organic aerosol episodes and quantitation of primary and secondary organic aerosol concentrations during SCAQS. Atmos Environ 29:3527-3544.

Turpin BJ, Lim HJ. 2001. Species contributions to $PM_{2.5}$ mass concentrations: revisiting common assumptions for estimating organic mass. Aerosol Sci Technol 35:602-610.

Yang F, He K, Ye B, Chen X, Cha L, Cadle SH, et al. 2005. One-year record of organic and elemental carbon in fine particles in downtown Beijing and Shanghai. Atmos Chem Phys 5:1449-1457.

Supplemental Material, Table S1. BP changes (mmHg) and 95% CIs associated with IQR increases in major air pollutants at concentrations during the preceding 1 to 3 days before the BP measurement

Pollutant	Exposure metric	IQR	SBP[a]	DBP
PM_{10}, μg/m^3	1-day	66.0	1.29 (0.41, 2.18)**	1.12 (0.49, 1.76)**
	2-day	64.5	1.48 (0.39, 2.56)**	1.15 (0.37, 1.92)**
	3-day	45.7	1.40 (0.50, 2.29)**	1.05 (0.41, 1.68)**
$PM_{2.5-10}$, μg/m^3	1-day	42.3	1.08 (0.14, 2.02)*	1.01 (0.34, 1.69)**
	2-day	30.2	0.73 (-0.15, 1.60)	0.69 (0.06, 1.32)*
	3-day	35.6	1.16 (-0.12, 2.43)	0.92 (0.02, 1.83)*
$PM_{2.5}$, μg/m^3	1-day	51.2	1.08 (0.17, 1.99)*	0.96 (0.31, 1.61)**
	2-day	42.0	1.12 (0.97, 2.08)*	0.79 (0.10, 1.49)*
	3-day	31.3	1.14 (0.28, 2.00)**	0.90 (0.29, 1.51)**
CO, ppm	1-day	0.74	-0.08 (-1.20, 1.03)	0.15 (-0.65, 0.96)
	2-day	0.75	0.48 (-1.18, 2.15)	0.40 (-0.80, 1.60)
	3-day	0.70	0.54 (-1.21, 2.30)	0.11 (-1.14, 1.37)
NO_X, ppb	1-day	35.1	1.38 (0.19, 2.57)*	1.00 (0.14, 1.86)*
	2-day	25.5	0.60 (-0.32, 1.51)	0.92 (0.27, 1.57)**
	3-day	22.5	0.33 (-0.65, 1.32)	0.79 (0.09, 1.49)*
NO_2, ppb	1-day	14.2	0.62 (-0.70, 1.95)	0.73 (-0.22, 1.69)
	2-day	12.8	0.75 (-0.62, 2.13)	1.45 (0.47, 2.43)**
	3-day	10.3	0.45 (-0.96, 1.86)	1.01 (0.02, 2.01)*
NO, ppb	1-day	26.8	1.51 (0.33, 2.68)*	0.97 (0.12, 1.82)*
	2-day	19.5	0.58 (-0.34, 1.50)	0.80 (0.14, 1.45)*
	3-day	17.2	0.31 (-0.68, 1.30)	0.74 (0.04, 1.44)*

*$P<0.05$, **$P<0.01$.

[a] Estimates are adjusted for age, BMI, season, month, day-of-study, squared day-of-study, day-of-week, hour-of-day, study site, and temperature and relative humidity in linear and quadratic terms.
